# The Influence of the Hydrophobic Polymeric Coating on 5-ASA Release from the Bipolymeric Milibeads with Amidated Pectin

**DOI:** 10.3390/ma14143924

**Published:** 2021-07-14

**Authors:** Dorota Wójcik-Pastuszka, Kinga Barczyszyn, Witold Musiał

**Affiliations:** Department of Physical Chemistry and Biophysics, Faculty of Pharmacy, Wroclaw Medical University, ul. Borowska 211A, 55-556 Wroclaw, Poland; dorota.wojcik-pastuszka@umed.wroc.pl (D.W.-P.); kinga.barczyszyn@gmail.com (K.B.)

**Keywords:** pectin, 5-ASA, kinetics, drug release

## Abstract

The industrial polymeric carriers for peroral mesalazine application exploit, i.a., cellulose or polyacrylic acid derivatives, polyvinylpyrrolidone, and modified starch. Pectins, as natural polymers, are interesting materials in pharmaceutical applications due to properties such as non-toxicity, biocompatibility, and biodegradability. The aim of the study was the evaluation of the release of the drug from coated pectin beads doped with synthetic polymers as drug carriers to the colon, as well as interactions between ingredients. The drug release was carried out using basket apparatus. The amount of 5-ASA (5-aminosalicylic acid, mesalazine) released to the pH = 7.4 buffer with pectinase was measured at selected time intervals using UV-Vis spectroscopy. The zero-, first-, and second-order kinetics, as well as Higuchi, Korsmeyer–Peppas, and Hixon–Crowell equations, were used to analyze the release pattern. The interactions between beads components were investigated employing FTIR spectrophotometry and DSC study. The dissolution of the drug was divided into two parts. It was found that the release of 5-ASA followed mainly the Higuchi equation. The mass transport in the first stage of the release followed a non-Fickian model and the parameter n was in the range of 0.74 ± 0.2–0.99 ± 0.2. The formulation doped with PA (polyacrylic acid) was the most appropriate and capable of overcoming the variable conditions of the gastrointestinal tract.

## 1. Introduction

Pectin is a natural polysaccharide occurring in the walls of plants [1]. The amount, structure, and chemical composition of pectins varies among plants, their parts, and over time [2]. A pectin molecule consists mainly of homogalacturonan. The structure contains linear 1,4-linked αD-galacturonic acid; some of the carboxyl groups are partially methyl-esterified at C-6 and acetyl-esterified at positions O-2 and/or O-3. Pectins were divided into two groups: high-methoxy pectins (HM-pectins) with more than 50% of all the galacturonic acid esterified and low-methoxy pectins (LM-pectins) with less than 50% of all the galacturonic acid esterified [3]. Pectin forms a stable gel via ionotropic gelation. According to this process, calcium ions and the oppositely charged carboxyl groups of the galacturonic acid initiate cross-linking [4]. The most stable gels were obtained in an acidic environment. The increasing pH to the value of 5.35 or higher caused the degradation and inability of the pectin solution to gel [5,6].

A modified form of pectin is amidated pectin. Its chain contains some of the galacturonic acid molecules changed using ammonia to a carboxylic acid amide. The in vitro and in vivo study showed that pectins and amidated pectins were degraded in the upper parts of the digestive tract, although they were fermented by intestinal microbiota during their passage through the large intestine in animals and humans [7]. In plants, pectin was degraded by the enzymes pectinase and pectinesterase during fruit ripening [8].

Pectin shows biocompatibility and biodegradability and therefore was used in medicine and pharmacy. It has antimicrobial and antiviral properties, decreases water solubility, and improves mechanical properties. It is a very good biomaterial for drug carriers, antimicrobial films, and coatings. It is also a good material for hydrogels, films, scaffolds, and nanoparticles formations [1]. It was found that pectin prevents colon cancer as a dietary fiber [9]. As a natural product, it is compatible with many drugs and harmless. The ability of the gel formation in the acidic environment and the dissolution in basic pH make pectins a suitable candidate for the drug delivery system to the colon. Moreover, pectin is resistant to the digestive enzymes of the intestines; therefore, it is a promising compound for drug administration to the colon. Pectin gels swell in the acidic environment of the stomach and release a small amount of the drug. However, in the case of the drug transportation to the colon, this ability ensures that the entire dose of the drug is delivered [1].

Slavutsky et al. [10] revealed that the pectin gel doped with Brea gum forms a strong gel. The DSC and FTIR studies show a strong interaction between functional groups of both polymers. It was observed that swelling and erosion of the xerogels were depended on the pH values. In addition, the release of the drug from the bipolymeric carrier can also be dependent on the pH value [10].

The aim of the study was to obtain coated pectin beads doped with various synthetic polymers for colon targeted drug delivery systems and evaluate the kinetics of the drug-mesalazine (5-ASA) release from the formed carriers. The cores of coated beads consisted of a natural polymer–pectin and a doped synthetic polymer and were named bipolymeric milibeads. Additionally, the interaction between polymers and the drug with polymers was carried out.

## 2. Materials and Methods

Amidated pectin (APN) was supplied from C&G (Jasło, Poland). Calcium chloride and tris(hydroxymethyl)aminomethane were obtained from Chempur (Piekary Śląskie, Poland). Hydrochloric acid was bought in StanLab (Lublin, Poland). Polyacrylic acid (PA) was delivered from Lubrizol (Brumach, Belgium) and ammonium acryloyldimethyltaurate (AX) from CLARIANT (Muttenz, Switzerland). Polyethylene glycol (PEG) 4000 was a gift from the local industry Hasco-Lek (Wrocław, Poland). Thirty percent dispersion of polyvinyl acetate stabilized with polyvinylpyrrolidone and sodium lauryl sulfate (PVAC-D) was supplied from BASF (Ludwigshafen, Germany). A blend of polyvinyl acetate and povidone in the ratio 8:2 (PVAC-P) was supplied from BASF (Ludwigshafen, Germany). The 5-aminosalicylic acid (5-ASA) was obtained from Sigma–Aldrich (St. Louis, MO, USA) and pectinase was bought in BROWIN (Łódź, Poland). All reagents were of analytical grade and were applied without additional purification.

### 2.1. Preparation of Coated Pectin Beads

The pectin beads were obtained by applying the ionotropic gelation method [11]. According to the process, the bond between negatively charged pectin carboxyl groups and positive calcium ions was formed, and the obtained gel was more stable. The composition of coated pectin beads was presented in Table 1. The procedure of the dry pectin beads preparation was described in our previous work [12]. Components of formulations were mixed with water using the homogenizer (CAT X120, Ballrechten-Dottingen, Germany) with the rotation speed of 20,000 rpm up to the homogenous solution was obtained. The concentration of 5-ASA was about 2.13% (*w*/*v*) in formulations F1–F5 and APN 4.3% (*w*/*v*) in formulations F1 and F6. In formulations F2–F5 and F7–F10, the amount of APN was 3.6% (*w*/*v*), and the synthetic polymer was approximately 0.7% (*w*/*v*). The solution was instilled through a needle (20 G) into a 2.0% solution of calcium chloride employing the peristaltic pump (PS-16-Sipper-Pump, PG-Instruments-Limited, Leicestershire, London, UK) with a flow speed of 2.0 mL/min. Calcium chloride solution was placed on the magnetic stirrer (2 Mag, magnetic emotion, Munich, Germany) with a rotation speed of 200 rpm. The obtained pectin beads were removed from the solution, washed with water, and dried at the temperature of 50 °C until the difference in mass between the measurements performed within 30 min was less than 0.5 mg [13]. The dried beads were coated using PVAC-D dispersion that was diluted with water 1:1 (*v*/*v*). The obtained dispersion was sprayed on the beads. The beads were dried and mixed at the temperature of 50 °C for about 4 h. The spraying and drying procedure was repeated three times. Finally, the mass of coated beads was determined. The coating mass deposited on the milibeads was calculated as a difference between uncoated and coated milibeads.

### 2.2. Morphological Study

The microscopic investigation was carried out employing a microscope (Motic-Mlc-150-C, Merazet, Poznań, Poland) coupled with a Moticam camera. The surface area of about 60 beads of each formulation was measured using the dedicated software Motic Plus 2.0 (Motic, Poznań, Poland, v.2.0). The microscope magnification was fixed to 7.5. The microscopic images of 60 weighted beads were recorded. The program calculated the area of each bead separately, and the mean value was calculated. The mean surface area per mean mass of all formulations, as well as the standard deviation (SD), were calculated, applying the above-presented procedure.

### 2.3. Carrier Stability Study

The stability of the carriers of F6–F10 was investigated in buffer solutions with pH = 1.0; 6.0 and 7.4 with pectinase. The sample of the dry formulation was weighed, placed in a sieve, and immersed in a pH = 1.0 buffer for 2 h. The beads were removed from the solution, dried with a paper towel at defined time intervals, and the mass was measured using an analytical balance (WAS 160/C/2, Radwag, Radom, Poland). After 2 h of the test, the beads were removed from pH = 1.0 buffer and placed in pH = 6.0 buffer for the next 2 h, and the weighing procedure was repeated. Finally, after 4 h from the beginning of the test, the beads were placed in pH = 7.4 buffer with pectinase and weight every 15 min in the same way as previously. Each formulation F6–F10 was studied twice to calculate the value of m_t_^av^/m_0_^av^, where m_t_^av^ was the average mass of the sample at selected time point t and the m_0_^av^ was the average mass of the sample at the beginning of the study. The standard deviation (SD) was calculated as well.

### 2.4. Release Study of 5-ASA from Coated Pectin Beads

The release of 5-ASA from formulations F1–F5 was carried out employing the dissolution apparatus 1 (ERWEKA, DT 126/128 light, Heusenstamm, Germany) [14]. The temperature of the test was 37 °C ± 0.5 °C, and the rotation speed of baskets was set at 50 rpm. In the first stage of the study, 5-ASA was released to the acceptor fluid with pH = 1.0 within 2 h. The samples of 3 mL were taken at defined time intervals, and the fresh acceptor fluid was added. Six tests were performed for each formulation. The spectrum of 5-ASA in the pH = 1.0 buffer was recorded, and the maximum absorbance at 302.5 nm was noticed. At this wavelength, the absorbance of samples was measured. The amount of the drug was calculated based on the calibration curve. In the second stage of the study, the drug was released to the buffer with pH = 7.4 with 5 mL of pectinase. The procedure was the same as in the case of the release of the drug to pH = 1.0 acceptor fluid. The maximum absorbance of 5-ASA was observed at 330 nm, and at this wavelength, the calibration curve was prepared. The absorbances of collected samples were measured. The samples with an absorbance above 1 were diluted. Based on the calibration curve, the concentration of each sample was calculated. The test was carried out within 8 h. The release of 5-ASA from formulations F1–F5 to pH = 7.4 buffer with pectinase was analyzed according to the Equation (1) zero-, Equation (2) first-, Equation (3) second-order kinetics as well as Equation (4) Higuchi, Equation (5) Korsmeyer–Peppas and Equation (6) Hixon–Crowell models [15,16,17]. The equations describing the process are as follows:

(1) $$m_{t}=m_{b}+k_{0}t$$

(2) $$\ln\left(m_{0}-m_{t}\right)\;=\;\ln\left(m_{0}\right)-k_{1}t$$

(3) $$\;\frac{1}{\left(m_{0}-m_{t}\right)}=\frac{1}{m_{0}}-k_{2}t$$

(4) $$m_{t}=k_{H}t^{0.5}$$

(5) $$\log\left(\frac{m_{t}}{m_{\infty }}\right)=\log k_{K-P}+n\log t$$

(6) $$m_{0}{}^{1/3}-m_{\mathrm{left}}{}^{1/3}=k_{H-C}t$$

where m_t_ is the amount of the drug released in time t; m_b_ is the amount of the drug in the solution before the release (usually it was 0); m_0_ is the amount of the drug in the formulation before the dissolution; m_∞_ is the amount of the drug released after the infinitive time (in this research after 8 h); m_left_ is the amount of the drug left in the formulation in time t; k_0_, k_1_, k_2_, k_H_; k_K−P_, k_H−C_ mean the zero-, first-, second-order, Higuchi, Korsmeyer–Peppas and Hixon–Crowell release rate constants, respectively; n is the parameter indicative of the drug release mechanism. The comparison between the release profiles was carried out based on the values of the difference factor f_1_ and the similarity factor f_2_ (Equations (7) and (8)):

(7) $$f_{1}=\frac{\sum_{t=1}^{n}\left|R_{t}-T_{t}\right|}{\sum_{t=1}^{n}R_{t}}\times 100$$

(8) $$f_{2}=50\times \log\left\{{\left[1+\frac{\sum_{t=1}^{n}{\left(R_{t}-{\;T}_{t}\right)}^{2}}{n}\right]}^{-0.5}\times 100\right\}$$

where n is the number of time points, R_t_ is the released value of the reference batch at time t, and T_t_ is the released value of the test batch at time t [18].

### 2.5. Statistical Analysis

The obtained results of the surface area per mass of beads were shown as the means and ± SD. The statistical properties of the surface area per mass of coated pectin beads F1–F10 were performed based on the one-way analysis of variance (ANOVA) method and Honestly Significant Difference test (Tukey’s HSD), and a *p*-value of 0.05 was taken as the minimum level of significance. The comparison between the dissolution curves was also conducted employing ANOVA and Tukey’s HSD test on a 95% confidence level [19].

### 2.6. FTIR Study

FTIR spectra of F1–F10 beads, as well as the corresponding physical mixtures, were recorded using FTIR spectrometer (Thermo Scientific Nicolet iS50, Waltham, MA, USA) coupled to ATR mode (Thermo Scientific Nicolet iS50, Waltham, MA, USA) in the wavelength range of 500–4000 cm^−1^. The spectra were an average of 32 scans at a resolution of 4 cm^−1^. The recorded data were analyzed using a dedicated computer program OMNIC Specta 2.0 (Thermo Scientific, Waltham, MA, USA, v.2.0).

## 3. Results and Discussion

### 3.1. Morphological Study

**Figure 1:**
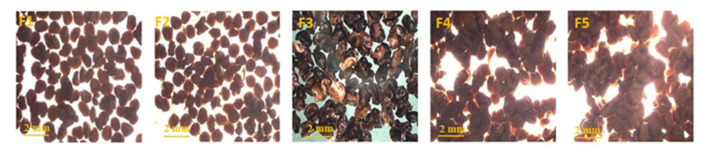
The microscopic images of coated pectin beads F1–F5.

The images of the obtained coated formulations F1–F5 loaded with 5-ASA were shown in Figure 1. Not many differences in the morphology between the preparations were noticed.

**Figure 2:**
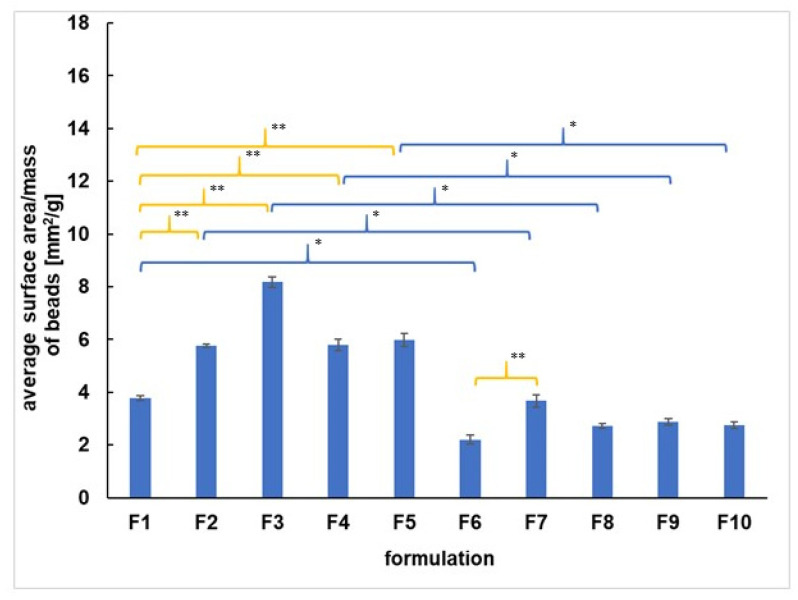
The average surface area per mass of coated beads F1–F10. Significant differences between the average values of surface area per mass of F1–F10 were marked, *p* < 0.05; * indicates the differences connected with 5-ASA incorporation, ** shows the discrepancies resulting from incorporation of the synthetic polymer.

The average surface area per mass of all formulations was analyzed, and the results were presented in Figure 2. It was found that the incorporation of 5-ASA into the beads resulted in changing the value of the surface area per mass in the case of all formulations studied. The surface area per mass of F1–F5 beads loaded with the drug was in the range from 3.8 ± 1.0 to 8.2 ± 4.2 mm^2^/g and was higher than the surface area per mass of formulations F6–F10 unloaded with 5-ASA, which was in the range from 2.2 ± 1.1 to 3.7 ± 2.6 mm^2^/g. The incorporation of synthetic polymers such as PA, PEG, AX, PVAC-P to the beads loaded with 5-ASA affected the value of the surface area per mass. The value of the surface area per mass of F2, F3, F4, F5 was higher than the value obtained for the F1 formulation (3.8 ± 1.0 mm^2^/g) not containing the synthetic polymer. Moreover, the incorporation of PA to unloaded with 5-ASA beads (F7) increased the surface area per mass in comparison to the value obtained for F6 not doped with the synthetic polymer. Additionally, the discrepancies between the surface area per mass of beads containing different synthetic polymers were revealed. The surface area per mass of F3 with PEG was 8.2 ± 4.2 mm^2^/g, and it was higher than the surface area per mass of F4 and F5 containing PA, AX, and PVAC-P. The values obtained for F2, F4, and F5 were similar, and they were in the range from 5.8 ± 1.2 to 6.0 ± 3.6 mm^2^/g. However, in the case of unloaded beads, the surface area per mass of F7 doped with PA was the highest among formulations F7–F10. The results of the surface area per mass of the beads study indicated that the addition of the second polymer PA, AX, or PVAC-P to the loaded with 5-ASA beads provoked the formation of more compact beads than the formulation doped with PEG. In the case of beads unloaded with the drug, the most compressed formulations were doped with PEG, AX, and PVAC-P as well as formulation without the synthetic polymer. A similar observation was found in Calvo et al.’s studies [20]. It may suggest that the chains of synthetic polymers interact with 5-ASA or/and with APN or/and with coating. The interaction between the COO- group from PA and the COO- group belonging to the drug, as well as the bond between PA and APN, were reported in our previous works [12,21].

The incorporation of synthetic polymers, as well as 5-ASA to the coated pectin preparations, influenced the value of the surface area per mass of the beads studied.

### 3.2. Carrier Stability Study

**Figure 3:**
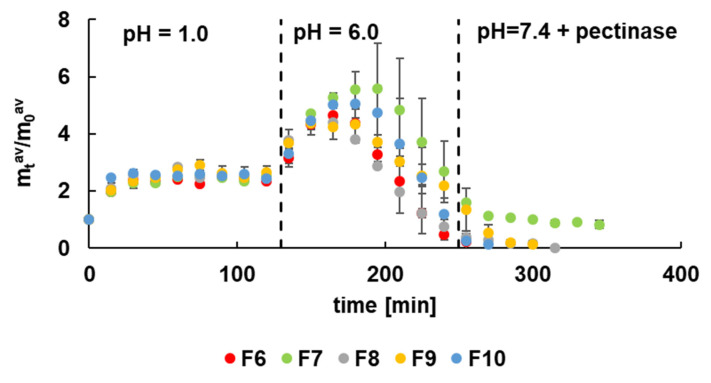
The dependence of the carrier mass of formulations F6–F10 in time at different pH.

The carrier stability investigation was performed at pH = 1.0, simulating the stomach environment, pH = 6.0 that reflect intestines conditions, as well as at pH = 7.4 with pectinase reflecting the colon environment. The obtained results were presented in Figure 3. It was noticed that the mass of formulations F6–F10 unloaded with 5-ASA immediately increased when introducing the beads into solutions at pH = 1.0. It was related to the swelling of the beads and the penetration of the solution into the carrier interior. After about 15 min, the equilibrium was established, which was maintained for the entire time the beads were in the acidic environment. It was interesting that at pH = 6.0, there was a further increase in the mass of the carrier, possibly caused by penetration of the solution. After reaching the maximum, a decrease in the mass of the carrier was observed due to the slow decomposition of the biopolymer. The increase of pH to 7.4 and the addition of the enzyme caused the pectin chains decomposition. In this environment, the glycosidic bonds of pectin broke down. The fastest degradation of the carrier was noticed in the case of F6 that practically decomposed in pH = 6.0. It was worth mentioning that the F6 formulation was not doped with the synthetic polymer. The formulations of F7–F10 decomposed slower, and the slowest was the degradation of F7. This observation can be explained by the addition of synthetic polymer to the beads that prolonged the stability of the carrier. Moreover, the coating also affected the carrier stability. By comparing the presented results with the observation from our previous work [12], it was found that uncoated beads not containing the synthetic polymer decomposed faster. The coated formulations doped with synthetic polymers, particularly with PA, decayed slower, and beads F7 did not disintegrate completely, as was shown in Figure 3.

Based on these results, it can be concluded that the most promising formulation was F7 doped with PA. It is consistent with the observation from the morphological study. The incorporation of PA to F2 gave more compact beads that were more stable and could overcome the variable conditions of the digestive tract.

### 3.3. Release Study of 5-ASA at Acidic Conditions

**Figure 4:**
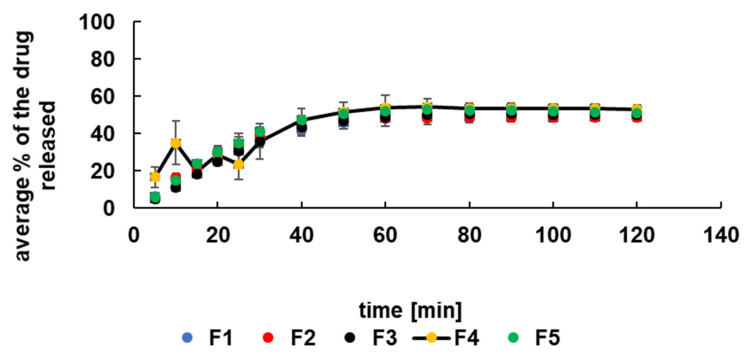
The release profiles of 5-ASA from F1–F5 in pH = 1.0 buffer.

The dissolution curves of the drug released to the pH = 1.0 solution were presented in Figure 4. It was observed that within 60 min about 50% of the drug was released from all formulations, and after this time, the equilibrium was reached. The release profiles were similar for all formulations studied. However, the dissolution of the drug from formulation F4 containing AX at the beginning of the process was slightly different. It was noticed that during the time period of 30 min, the amount of the drug released increased and decreased. It may be connected with the incorporation of synthetic polymer. AX is a bifunctional copolymer containing an acidic and basic group in its structure. It was estimated that at the time of preparation of the F4 formulation, the value of pH was about 1.5. French et al. [22] reported that the solubility of 5-ASA increased at pH < 2.0 and pH > 5.5 and was the lowest in the pH range from 2.0 to 5.5. The estimated pH of F4 beads favors the high solubility of 5-ASA. The decrease of pH to 1.0 level in the presence of acceptor HCl solution may positively influence the increase in 5-ASA concentrations in the acceptor fluid. Moreover, 5-ASA is an amphoteric molecule with pK_a1_ (carboxyl) = 2.30 ± 0.09 and pK_a2_ (amino) = 5.69 ± 0.04 [22]. Aunis et al. [23], in their research, proposed a model of the dissolution of the drug in the pH-changing solution. At low pH values, the amphoteric molecule reacts with acid species producing the positively charged drug molecule. When the pH of the solution increases, 5-ASA reacts with the buffering medium giving the negatively charged ionized form. According to French et al. [22], the drug may affect local changes in the pH value. In the present study, 5-ASA may be bounded by the polymer decreasing the release to the acceptor fluid. On the other hand, the microenvironmental changes in the pH value may influence the solubility of 5-ASA. The established acid-base balance in the formulation of F4 was disturbed when beads were placed in the solution, and the specific behavior was observed (Figure 4). In the case of PA, an anionic polymer, the dissociation of the carboxyl groups in the acidic conditions was hindered, and F2 beads acted similarly to formulations with PEG 4000 and PVAC-P, which are nonionic polymers. The release of the drug from all formulations studied may also be connected with the swelling process of the beads that were observed in Figure 3. Beads structure became looser during swelling and the drug more easily diffused to the solution.

### 3.4. Kinetics of 5-ASA Release at pH = 7.4 with Pectinase

The maximum percent of the drug released to the pH = 7.4 buffer with pectinase was 71.0 ± 2.0% and was obtained from beads F5. Next to this was observed for the release of the drug from F4, and it was 69.0 ± 7.0%. The percent of the drug released from the F1 formulation was 67.0 ± 2.0% and from F3 was 65.0 ± 4.0%. The lowest amount of the drug released was 44.0 ± 12.0%, observed in the case of the dissolution of 5-ASA from F2 formulation. It was consistent with results obtained from the carrier stability study. The most stable beads in the colon environment were beads F2 doped with PA.

**Figure 5:**
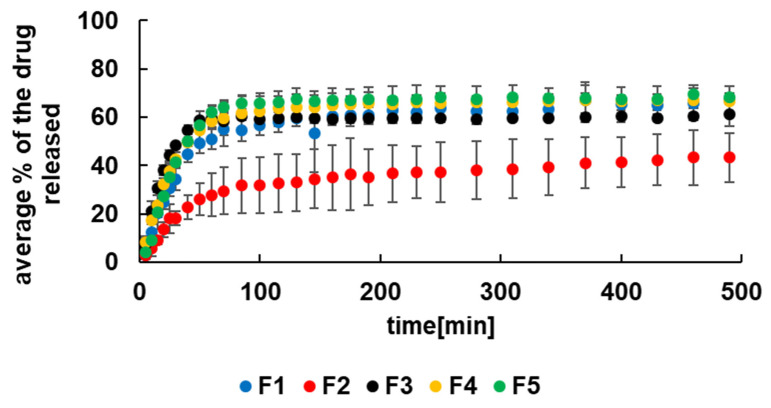
The release curves of 5-ASA from formulations F1–F5 to pH = 7.4 buffer with pectinase.

Based on the results presented in Figure 5, it can be said that the system does not release 100% of the drug in the applied conditions. After the release of ca. 60% of the 5-ASA, the system achieved equilibrium, and the remaining 5-ASA resided in the formulation, presumably bonded with the polymeric chain.

The estimated pH of F2 beads was about 3.0 that favors the low solubility of 5-ASA. The increasing pH in the buffer solution to 7.4 causes an increase in the drug concentration in the acceptor medium [22]. Additionally, the amphoteric molecule of 5-ASA may bound to the incorporated anionic polymer-PA that in the slightly alkaline conditions was in the anionic form.

The drug release patterns of formulations F1–F5 to the pH = 7.4 buffer with pectinase were presented in Figure 5. It was noticed that the dissolution of 5-ASA was very fast at the beginning and slowed down in the next step of the process. It was found that none of the employed kinetic models was appropriate to describe the whole dissolution process. In order to calculate the kinetic parameters, the release process was divided into two stages. The first stage was from the beginning to 70 min and the second stage was from 70 min to the end of the measurement. It was worth mentioning that the first stage of the dissolution was a complex process and was also connected with the swelling of the beads.

The kinetic release parameters were determined for both stages of the dissolution and were listed in Table 2 and Table 3. It was revealed that all employed kinetic models were appropriate to characterize the release of 5-ASA from formulations F1–F5 in the first stage. All values of linear correlation coefficient (R^2^) obtained for the first release stage were in the range from 0.83 ± 0.04 to 0.99 ± 0.01. However, the first stage of the drug release from F1–F3 was best described by the Higuchi model. In the case of 5-ASA release from F4 and F5, the second-order kinetics was more suitable. According to the Higuchi model, the drug was released the fastest from F3 with the rate constant 43.1 ± 3.6 mg × min^−1/2^ and a half release time of 40.06 ± 6.6 min. The lowest value of k_H_ and the highest of t_0.5_ were obtained for the release of the drug from F4. However, based on the results from the second-order equation, the lowest value of the rate constant k_S-O_ = (1.5 ± 0.2) × 10^−5^ mg^−1^ × min^−1^ and the highest value of t_0.5_ = 187.3 ± 39.1 min were calculated for the dissolution of 5-ASA from F2. Additionally, the lowest value of the rate constant and the highest value of the half release time was obtained in the case of the drug release from F2 also using Z-O, F-O, H–C models.

**Figure 6:**
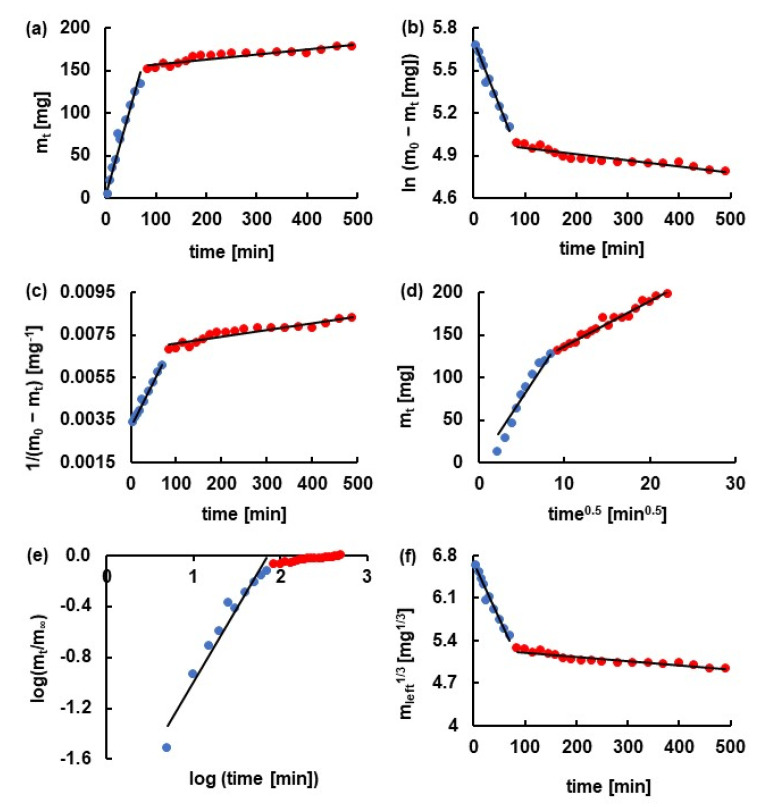
The kinetics of the release of 5-ASA from F2 formulation to pH = 7.4 buffer with pectinase according to (**a**) zero-order model; (**b**) first-order model; (**c**) second-order model; (**d**) Higuchi model; (**e**) Korsmeyer–Peppas model; (**f**) Hixon–Crowell model. Points mean experimental data, and the black line was the theoretical curve. Blue points indicated the first stage of the release, and red points indicated the second stage of the release.

It is consistent with the results presented in Figure 5. The release curve of 5-ASA from F2 doped with PA was situated significantly lower than the profiles obtained for the dissolution of the drug from F1, F3–F5. Moreover, the beads of F7 doped with PA and unloaded with 5-ASA were the most stable in the colon environment (Figure 3). It was worth mentioning that the highest values of the rate constants and the lowest values of the half release time were determined mainly in the case of the release of 5-ASA from F5 (Z-O, F-O, S-O, H–C model). Only in the case of the dissolution of the drug from F3 using H and K–P models the highest values of the rate constant and the lowest of the half release time were obtained. These results indicated that the incorporation of a synthetic polymer not always prolonged the dissolution. The changing of the release pattern and the kinetic parameters depend on the type of synthetic polymer incorporated into the beads. The example of fitting the experimental points to the theoretical curves was presented in Figure 6. It was revealed that the value of the parameter n from the K–P equation obtained from the release of 5-ASA from F1–F5 was in the range of 0.74 ± 0.2–0.99 ± 0.2, meaning the mass transfer following a non-Fickian model. Peppas concluded in his study that the value of n between 0.5 and 1.0 indicated anomalous transport [24]. In the case of the release of 5-ASA from uncoated beads, the values of n were in the range from 0.30 ± 0.07 to 0.50 ± 0.07, meaning a Fickian diffusion mechanism. Only in the case of the release of 5-ASA from uncoated beads doped with PA the parameter of n was 0.65 ± 0.8 [12]. The obtained results in the present work suggest that the coating changes the mass transport mechanism.

Costa et al. [17] and Suvakanta et al. [25] found in their study that to the determination of the parameter n, only the portion of the release curve where m_t_/m_∞_ < 0.6 may be used. In the present investigation, this portion was included in the first stage of the dissolution curve, and neither n value nor the other kinetic parameters on the basis of the K–P model for the second stage release study were determined. The kinetic parameters obtained for the second stage of the dissolution employing other kinetic models were presented in Table 3.

The values of linear correlation coefficients calculated for the second stage of the release were in the range from 0.80 ± 0.01 to 0.91 ± 0.05 and were slightly lower than in the first stage of the dissolution. The best fit was obtained using the Higuchi model describing the drug release from all formulations studied apart from F3. Based on this model, the drug was released the fastest from F2 with the release rate constants of 5.2 ± 0.8 mg × min^−1/2,^ and the half release time was 234 ± 83 min. The lowest value of k_H_ = 1.9 ± 0.2 mg × min^−1/2^ was obtained for the drug dissolution from F5, and t_0.5_ was 9304 ± 2776 min. Similar results were found in the case of the Z-O model. However, from S-O kinetics, the highest value of k_2_ was derived for 5-ASA release from F2, similar to the first stage and the lowest for the drug release from F4 beads. Analysing the obtained rate constants, it was noticed that in Z-O, F-O, H, H–C models, 5-ASA was released the slowest from F5, in contrast to the first step where the drug release rate constants from F5 were the highest in most cases. It should be mention that the variability between the kinetic parameters was not significant; values were of the same order of magnitude.

The main idea of the work was to study the release kinetics of the drug at pH = 7.4 in the presence of an enzyme, which reflected the colon environment. The stability investigations were performed in three different media, which reflected the pH of the consequent parts of the digestive tract environment. The study revealed that the beads might overcome the variable pH conditions of the gastrointestinal tract and may reach the large intestine. The dissolution plots in low pH were obtained additionally to gain more information on the drug release during beads swelling.

### 3.5. The Difference Factor f_1_ and the Similarity Factor f_2_

In order to compare the release curves of 5-ASA from formulations F1–F5, the difference factor and the similarity factor were calculated. FDA recommended that the dissolution profiles are similar if the values of f_1_ are close to 0, usually are in the range 0–15. The values of f_2_ close to 100, usually from the range from 50–100, indicate sameness or equivalence of the compared profiles [18]. In the presented study, the obtained results of f_1_ and f_2_ were summarised in Table 4. It was found that the differences were between the drug release pattern from F1 and F2. The value of the difference factor was higher than 15 (41.0), and the value of the similarity factor was below 50 (32.7). However, in the comparison of the release curve of F1 with the remaining profiles, the obtained values of f_1_ and f_2_ did not indicate discrepancies. It should be noticed that the differences were also observed between the release profiles of F2 and F3, F4, F5. It can be concluded that the analysis of f_1_ and f_2_ parameters indicated the difference between the release of 5-ASA from F2 doped with PA and from the rest formulations. The incorporation of PA to the beads changed the release behavior.

### 3.6. Statistical Analysis

The release profiles of 5-ASA from formulations F1–F5 were analyzed employing ANOVA. It was found that there were statistically significant differences between groups. According to Tukey’s HSD test, the calculated value of HSD was 11.6, and the obtained values of differences between group means were listed in Table 5. The statistically significant differences were observed between F1–F2, F2–F3, F2–F4 as well as F2–F5 groups. Based on these data, the discrepancies between the dissolution curve of 5-ASA from F2 and the remaining formulations were found. The results were consistent with outcomes from f_1_ and f_2_ calculations.

### 3.7. FTIR Study

**Figure 7:**
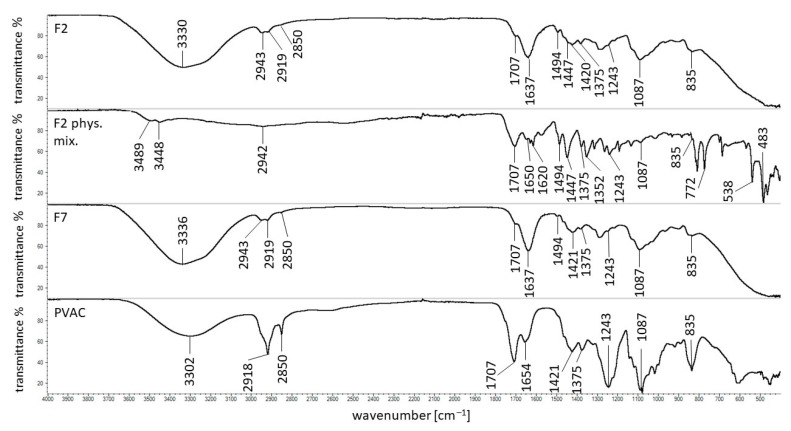
FTIR spectra of formulation F2, its physical mixture, F7, and PVAC-D.

The spectroscopic studies of pure ingredients of beads, uncoated formulations loaded and unloaded with the drug, as well as their corresponding physical mixtures, were carried out in our previous work [12]. In the present work, the investigation was mainly focused on the interaction between the polymer, PVAC-D, used as a coating, and other formulation ingredients. As an example, the FTIR spectra of formulation F2, its physical mixture, formulation F7, and PVAC-D were shown in Figure 7. The spectra of formulations F1, F3–F6, and F8–F10, as well as the corresponding physical mixtures, were presented in Figures S1–S4 in Supplementary Materials. In the spectrum of PVAC-D, the broad band was observed at 3302 cm^−1^ that may be connected with the presence of the water molecule. The other maxima were recorded at 2918 and 2850 cm^−1^, the characteristic stretching vibration belonging to the C=O group was noticed at 1707 cm^−1^, and the band located at 1243 cm^−1^ was assigned to the C–O group. Additionally, the signals at 1654, 1421, 1375, 1087, and 835 cm^−1^ were also noticed, and the observations were in good agreement with the results obtained by D’Amelia et al. [26] and Wei et al. [27]. It was revealed that all the bands were found in the spectra of F1–F10 formulations as well as in their physical mixtures apart from the band at 1654 cm^−1^ that was shifted to 1637 cm^−1^ in the spectra of F1–F10. Only in the case of the F5 spectrum, the band at 1637 cm^−1^ was not observed. In this region, the bands at 1650 and 1617 cm^−1^ belonging to 5-ASA were found. This observation may suggest the interaction between PVAC-D and APN that was present in all formulations F1–F10. Additionally, it was revealed that the maximum of APN at 1736 cm^−1^ present in all uncoated beads disappeared in the spectra of formulations of F1–F10 [12]. Formulations F6–F10 were unloaded with 5-ASA, and the interaction between the coating and the drug may be excluded.

The new band observed at 1637 cm^−1^ may also be connected with the interaction between the coating and the synthetic polymers. The band of pure PA at 1697 cm^−1^ was not found in the spectra of uncoated beads nor in the spectra of coated beads, indicating the possibility of the interaction of PA with APN as well as with PVAC-D. This observation was consistent with the results obtained in the stability study (Figure 3) and may explain the different behavior of formulation doped with PA in comparison to the rest formulations. The most stable formulation that can overcome pH = 1.0; 6.0 and 7.4 with pectinase was coated F7 doped with PA.

The interaction between PA and PVAC-D may also explain the differences in the release of 5-ASA from F2 comparing the dissolution of the drug from F1. The statistical analysis indicated the discrepancies in the release of 5-ASA from F2 and from the rest formulations as well. Sahariah et al. [28] studied the interaction between two carbonyl groups. It was found that there was a possibility to create a noncovalent bond between these molecules. The possibility of the bond formation between two oxygen atoms of carbonyl groups C=O…O=C was also studied by Fayzullin et al. [29]. All the above information may suggest the existence of the interaction between the carbonyl group of PA and the carbonyl group belonging to PVAC-D.

In the case of formulation F3 doped with PEG 4000, the signal of the synthetic polymer at 2881 cm^−1^ was found only in the spectrum of the F3 physical mixture but was not present in the spectra of F3 nor in F8. The observation was consistent with the result of the uncoated beads study in which the interaction between the hydroxyl groups of PEG 4000 and APN was postulated. The band at 1096 cm^−1^ belonging to PEG 4000 was noticed only in the spectrum of the physical mixture of F3 but was shifted to1076 cm^−1^ in the spectra of F3 and F8. However, it was difficult to assign the signal unambiguously to PEG 4000 because, at 1087 cm^−1^, the characteristic maximum of PVAC-D was observed. The appearance of a new band at 1076 cm^−1^ cannot exclude the bond formation between PEG 4000 and PVAC-D. Wu et al. [30] revealed the possibility of bond formation between the hydroxyl group and the carbonyl group of PVAC-P.

The FTIR spectra of F4, its physical mixture, and F9 were presented in Figure S3. The formulations were doped with AX, but the characteristic bonds at 1640, 1544, 1440, 1388, and 1176 cm^−1^ were not noticed, similar to the study of uncoated beads. Besides the interaction of AX with APN, the carbonyl groups of the synthetic polymer may also bind to the coating carbonyl group, and it was difficult to distinguish which molecule interacts with AX. It was also possible that both of them form a bond. The analysis of the FTIR spectra of F5, its physical mixture, and F10 gave analogous results. The characteristic bands of PVAC-P at 1731 and 1661 cm^−1^ were found in the spectrum of the F5 physical mixture at 1731 and 1673 cm^−1^, but both disappeared in the spectra of F5 and F10 formulations. The beads were incorporated with PVAC-P, which carbonyl groups may interact with carbonyl groups of APN as well as PVAC-D.

The maxima observed at the wavelength about 3489 and 3448 cm^−1^ in the spectra of physical mixtures indicated the presence of CaCl_2_, and they disappeared in the spectra of formulations F1–F10 because a chemical bond of Ca^2+^ with APN was formed. The spectroscopic study confirms that the coating of the beads may increase the stability of the formulation by a bond formation and ensure to reach the large intestine.

In our previous paper, we evaluated the uncoated beads, whereas, in the present work, we presented the results obtained for coated formulations. The further study will evaluate the most promising beads-doped with PA–coated with another polymer to obtain a more effective cessation of the release of the drug in the stomach at low pH. On the other hand, the limited release of mesalazine in the gastric fluid may be beneficial in some cases. The last decade has brought more and more information about the changes in the stomach and duodenum accompanying ulcerative colitis. The use of a mesalazine preparation in which a part of the active ingredient is released in the stomach may favorably influence the course of treatment of complex inflammatory lesions within GT [31,32,33,34,35,36,37,38]. The latest scientific articles link the inflammatory lesions in the duodenum or stomach with bowel inflammation [39], which suggests the regions of drug targeting.

## 4. Conclusions

The widely applied polymers used in the formulation of mesalazine are cellulose or polyacrylic acid derivatives, polyvinylpyrrolidone, and modified cellulose. In our research, the incorporation of synthetic polymers such as PA, PEG, AX, and PVAC-P into the beads loaded with 5-ASA resulted in an increase in the surface area per mass comparing to the surface area per mass of formulations not containing the synthetic polymer. However, the addition of the second polymer PA, AX, and PVAC-P to the beads loaded with 5-ASA provoked the formation of more compact beads in comparison to the formulation doped with PEG. The addition of PA to the beads, as well as the coating, prolonged the stability of the carrier in acidic conditions. The lowest amount of the drug released to the buffer of pH = 7.4 with pectinase was 44.0 ± 12.0% and was observed in the case of the dissolution of 5-ASA from F2 formulation doped with PA. The release process was divided into two stages. The drug release was well fitted to the Higuchi model and to the second-order kinetics. The obtained value of the parameter n from the K–P equation was in the range of 0.74 ± 0.2–0.99 ± 0.2, which indicates the non-Fickian model mass transfer. FTIR and DSC studies indicated the possibility of the bond formation between synthetic polymers and APN or coating polymers.

The conducted research revealed that the F2 formulation, doped with PA and coated with a synthetic polymer, was the most promising formulation capable of overcoming the variable conditions of the gastrointestinal tract and delivering the drug to the colon.

## Figures and Tables

**Table 1:** The composition of formulations F1–F10.

	F1	F2	F3	F4	F5	F6	F7	F8	F9	F10
5-ASA [g]	3	3	3	3	3	―	―	―	―	―
APN [g]	6	5	5	5	5	6	5	5	5	5
PA [g]	―	1	―	―	―	―	1	―	―	―
PEG400 [g]	―	―	1	―	―	―	―	1	―	―
AX [g]	―	―	―	1	―	―	―	―	1	―
PVAC-P [g]	―	―	―	―	1	―	―	―	―	1
PVAC-D [g]	1.5	1.3	1.3	1.6	1.6	1.5	1.5	1.4	1.4	1.3

**Table 2:** The kinetic parameters of the first stage of the release of 5-ASA from F1–F5.

Kinetic Model	Kinetic Parameters	F1	F2	F3	F4	F5
Z-O	k_0_ [mg × min^−1^]	4.7 ± 0.6	2.5 ± 0.3	6.2 ± 1.2	5.4 ± 0.8	5.4 ± 0.6
	t_0.5_ [min]	58.8 ± 6.8	106.6 ± 13.7	44.3 ± 8.6	47.2 ± 7.0	46.2 ± 5.1
	R^2^	0.96 ± 0.01	0.97 ± 0.02	0.92 ± 0.02	0.95 ± 0.02	0.97 ± 0.01
F-O	k_1_ × 10^2^ [min^−1^]	1.2 ± 0.2	0.5 ± 0.1	1.2 ± 0.4	1.3 ± 0.2	1.6 ± 0.2
	t_0.5_ [min]	60.1 ± 10.9	155.7 ± 34.9	56.5 ± 16.9	55.2 ± 10.0	43.6 ± 5.4
	R^2^	0.94 ± 0.03	0.92 ± 0.05	0.85 ± 0.03	0.94 ± 0.04	0.97 ± 0.01
S-O	k_2_ × 10^5^ [mg^−1^ × min^−1^]	3.8 ± 0.6	1.5 ± 0.2	4.2 ± 1.0	5.3 ± 0.5	5.8 ± 0.4
	t_0.5_ [min]	55.1 ± 8.4	187.3 ± 39.1	110.1 ± 27.0	46.2 ± 6.2	35.1 ± 2.6
	R^2^	0.95 ± 0.03	0.93 ± 0.05	0.89 ± 0.03	0.97 ± 0.02	0.99 ± 0.01
H	k_H_ [mg × min^−1/2^]	32.1 ± 3.4	17.3 ± 1.8	43.1 ± 3.6	5.4 ± 0.8	36.3 ± 4.4
	t_0.5_ [min]	63.0 ± 13.4	258.4 ± 49.8	40.06 ± 6.6	2238.7 ± 673.6	47.0 ± 11.6
	R^2^	0.97 ± 0.02	0.98 ± 0.01	0.98 ± 0.004	0.95 ± 0.02	0.97 ± 0.01
K–P	k_K-P_ × 10^2^ [min^−n^]	1.8 ± 1.1	2.0 ± 0.2	5.6 ± 2.3	3.6 ± 1.2	1.4 ± 0.7
	t_0.5_ [min]	33.0 ± 30.1	40.1 ± 25.7	20.3 ± 14.7	26.5 ± 17.0	30.2 ± 25.3
	R^2^	0.92 ± 0.03	0.95 ± 0.02	0.90 ± 0.09	0.95 ± 0.03	0.92 ± 0.1
	n	0.95 ± 0.20	0.87 ± 0.1	0.74 ± 0.2	0.80 ± 0.1	0.99 ± 0.2
H–C	k_H-C_ × 10^2^ [mg^1/3^ × min^−1^]	2.7 ± 0.5	1.3 ± 0.2	2.8 ± 0.9	2.9 ± 0.5	3.5 ± 0.5
	t_0.5_ [min]	61.9 ± 12.3	147.7 ± 34.1	60.6 ± 19.6	58.6 ± 11.9	46.6 ± 7.1
	R^2^	0.93 ± 0.03	0.91 ± 0.05	0.83 ± 0.04	0.93 ± 0.04	0.95 ± 0.02
best fit		H	H	H	S-O	S-O

Z-O—zero-order model; F-O—first-order model; S-O—second-order model; H—Higuchi model; K–P—Korsmeyer–Peppas model; H–C—Hixon–Crowell model.

**Table 3:** The kinetic parameters of the second stage of the release of 5-ASA from F1–F5.

Kinetic Model	kinetic Parameters	F1	F2	F3	F4	F5
Z-O	k_0_ [mg × min^−1^]	0.12 ± 0.02	1.6 ± 0.02	0.08 ± 0.02	0.08 ± 0.01	0.06 ± 0.08
	t_0.5_ [min]	1262 ± 258	525 ± 94	3246 ± 784	2186 ± 366	3019 ± 488
	R^2^	0.86 ± 0.04	0.89 ± 0.05	0.82 ± 0.08	0.88 ± 0.05	0.91 ± 0.05
F-O	k_1_ × 10^4^ [min^−1^]	6.3 ± 1.1	4.9 ± 0.8	4.0 ± 0.8	4.7 ± 0.7	3.9 ± 0.5
	t_0.5_ [min]	1168 ± 224	1431 ± 223	3077 ± 735	1562 ± 261	2070 ± 328
	R^2^	0.87 ± 0.04	0.90 ± 0.05	0.82 ± 0.09	0.89 ± 0.04	0.91 ± 0.05
S-O	k_2_ × 10^6^ [mg^−1^ × min^−1^]	3.3 ± 0.6	1.8 ± 0.3	2.1 ± 0.4	3.4 ± 0.5	2.5 ± 0.3
	t_0.5_ [min]	1423 ± 263	1790 ± 275	4152 ± 986	1990 ± 319	2715 ± 426
	R^2^	0.88 ± 0.04	0.90 ± 0.05	0.82 ± 0.09	0.90 ± 0.03	0.92 ± 0.05
H	k_H_ [mg × min^−1/2^]	3.9 ± 0.6	5.2 ± 0.8	2.4 ± 0.6	2.6 ± 0.4	1.9 ± 0.2
	t_0.5_ [min]	1422 ± 535	234 ± 83	15,552 ± 7072	4739 ± 1415	9304 ± 2776
	R^2^	0.89 ± 0.04	0.90 ± 0.06	0.80 ± 0.01	0.91 ± 0.03	0.92 ± 0.04
H–C	k_H-C_ × 10^3^ [mg^1/3^ × min^−1^]	1.2 ± 0.2	1.1 ± 0.2	0.8 ± 0.2	0.8 ± 0.1	0.7 ± 0.1
	t_0.5_ [min]	1090 ± 212	1337 ± 210	2720 ± 657	1352 ± 234	1928 ± 309
	R^2^	0.87 ± 0.04	0.90 ± 0.05	0.82 ± 0.07	0.89 ± 0.04	0.91 ± 0.05
best fit		H	F-O, S-O, H, H–C	Z-O, F-O, S-O, H–C	H	S-O, H

Z-O—zero-order model; F-O—first-order model; S-O—second-order model; H—Higuchi model; H–C—Hixon–Crowell model.

**Table 4:** The calculated values of the difference factor f_1_ and the similarity factor f_2_.

	f_1_				f_2_			
Formulation	F2	F3	F4	F5	F2	F3	F4	F5
F1	41.0	8.2	9.5	11.2	32.7	60.4	64.1	59.9
F2	―	79.3	85.9	88.0	―	30.3	28.5	27.5
F3	―	―	8.48	12.3	―	―	65.2	57.6
F4	―	―	―	4.1	―	―	―	76.2

**Table 5:** The calculated values of differences between group means.

Formulation	F2	F3	F4	F5
F1	21.2	2.9	4.9	5.6
F2	―	24.1	26.1	26.8
F3	―	―	2.0	2.6
F4	―	―	―	0.6

## Data Availability

The data presented in this study are available in presented article entitled: The Influence of the Hydrophobic Polymeric Coating on 5-ASA Release from the Bipolymeric Milibeads with Amidated Pectin and the attached Supplementary Material.

## Supplementary Materials

The following are available online at https://www.mdpi.com/article/10.3390/ma14143924/s1, Figure S1: FTIR spectra of formulation F1, its physical mixture, and F6, Figure S2: FTIR spectra of formulation F3, its physical mixture, and F8, Figure S3: FTIR spectra of formulation F4, its physical mixture, and F9, Figure S4: FTIR spectra of formulation F5, its physical mixture, and F10, Figure S5: The thermogram of formulation F1 (red line), F6 (blue line), and the physical mixture of F1 (black line) as well as the curve of PVAC-D (green line), Figure S6: The thermogram of formulation F2 (red line), F7 (blue line), and the physical mixture of F2 (black line), Figure S7: The thermogram of formulation F3 (red line), F8 (blue line), and the physical mixture of F3 (black line), Figure S8: The thermogram of formulation F4 (red line), F9 (blue line), and the physical mixture of F4 (black line), Figure S9: The thermogram of formulation F5 (red line), F10 (blue line), and the physical mixture of Figure 5 (black line), Table S1: The DSC endotherms and exotherms (↓) of formulations F1–F10 and the corresponding physical mixtures (M). The listed numbers refer to the value of temperature.

## References

[1] Martau G.A., Mihai M., Vodnar D.C. (2019). The Use of Chitosan, Alginate, and Pectin in theBiomedical and Food Sector-Biocompatibility, Bioadhesiveness, and Biodegradability. Polymers.

[2] Bidhendi A.J., Geitmann A. (2016). Relating the mechanics of the primary plant cell wall to morphogenesis. J. Exp. Bot..

[3] Kameshwar A.K.S., Qin W. (2018). Structural and functional properties of pectin and lignin–carbohydrate complexes de-esterases: A review. Bioresour. Bioprocess..

[4] Liang R.H., Chen J., Liu W., Liu C.M., Yu W., Yuan M., Zhou X.Q. (2012). Extraction, characterization and spontaneous gel-forming property of pectin from creeping fig (*Ficus pumila* Linn.) seeds. Carbohydr. Polym..

[5] Moreira H.R., Munarin F., Gentilini R., Visai L., Granja P.L., Tanzi M.C., Petrini P. (2014). Injectable pectin hydrogels produced by internal gelation: PH dependence of gelling and rheological properties. Carbohydr. Polym..

[6] Gawkowska D., Cybulska J., Zdunek A. (2018). Structure-related gelling of pectins and linking with other natural compounds: A review. Polymers.

[7] Mortensen A., Aguilar F., Crebelli R., Di Domenico A., Dusemund B., Frutos M.J., Galtier P., Gott D., Gundert-Remy U., Lambré C. (2017). Re-evaluation of pectin (E 440i) and amidated pectin (E 440ii) as food additives. EFSA J..

[8] Wang D., Yeats T.H., Uluisik S., Rose J.K.C., Seymour G.B. (2018). Fruit Softening: Revisiting the Role of Pectin. Trends Plant Sci..

[9] Zhang W., Xu P., Zhang H. (2015). Pectin in cancer therapy: A review. Trends Food Sci. Technol..

[10] Slavutsky A.M., Bertuzzi M.A. (2019). Formulation and characterization of hydrogel based on pectin and brea gum. Int. J. Biol. Macromol..

[11] Ahirrao S.P., Gide P.S., Shrivastav B., Sharma P. (2014). Ionotropic Gelation: A Promising Cross Linking Technique for Hydrogels. J. Pharm. Nanotechnol..

[12] Wójcik-Pastuszka D., Potempa A., Musiał W. (2020). Bipolymeric Pectin Millibeads Doped with Functional Polymers as Matrices for the Controlled and Targeted Release of Mesalazine. Molecules.

[13] (2018). European Pharmacopoeia 10.0.

[14] (2017). European Pharmacopoeia 9.0.

[15] Ritger P.L., Peppas N.A. (1987). A simple equation for description of solute release I. Fickian and non-fickian release from non-swellable devices in the form of slabs, spheres, cylinders or discs. J. Control. Release.

[16] Siepmann J., Peppas N.A. (2011). Higuchi equation: Derivation, applications, use and misuse. Int. J. Pharm..

[17] Costa P., Lobo J.M.S. (2001). Modeling and comparison of dissolution profiles Paulo. Eur. J. Pharm. Sci..

[18] (1997). US Department of Health and Human Services Food and Drug Administration Center for Drug Evaluation and Research, Guidance for Industry Dissolution Testing of Immediate Release Solid Oral Dosage Forms. http://www.fda.gov/downloads/Drugs/.../Guidances/ucm070246.pdf.

[19] O’Hara T., Dunne A., Butler J., Devane J. (1998). A review of methods used to compare dissolution profile data. Pharm. Sci. Technol. Today.

[20] Aguirre Calvo T., Santagapita P. (2016). Physicochemical Characterization of Alginate Beads Containing Sugars and Biopolymers. J. Qual. Reliab. Eng..

[21] Wójcik-Pastuszka D., Mazurek K.L., Szumny A.J., Alagöz F., Musiał W.S. (2017). Properties of pectin based polymeric matrices for targeted drug delivery. Acta Pol. Pharm. Drug Res..

[22] French D.L., Mauger J.W. (1993). Evaluation of the Physicochemical Properties and Dissolution Characteristics of Mesalamine: Relevance to Controlled Intestinal Drug Delivery. Pharm. Res. An Off. J. Am. Assoc. Pharm. Sci..

[23] Aunis J.G., Southard M.Z., Myers R.A., Himmelstein K.J., Stella V.J. (1985). Dissolution of Carboxylic Acids III: The Effect of Polyionizable Buffers. J. Pharm. Sci..

[24] Peppas N.A. (1985). Analysis of Fickian and non-Fickian drug release from polymers. Pharm. Acta Helv..

[25] Dash S., Murthy P.N., Nath L., Chowdhury P. (2010). Kinetic modeling on drug release from controlled drug delivery systems. Acta Pol. Pharm..

[26] D’Amelia R.P., Gentile S., Nirode W.F., Huang L. (2016). Quantitative Analysis of Copolymers and Blends of Polyvinyl Acetate (PVAc) Using Fourier Transform Infrared Spectroscopy (FTIR) and Elemental Analysis (EA). World J. Chem. Educ..

[27] Wei S., Pintus V., Schreiner M. (2012). Photochemical degradation study of polyvinyl acetate paints used in artworks by Py-GC/MS. J. Anal. Appl. Pyrolysis.

[28] Sahariah B., Sarma B.K. (2019). Relative orientation of the carbonyl groups determines the nature of orbital interactions in carbonyl-carbonyl short contacts. Chem. Sci..

[29] Fayzullin R.R., Shteingolts S.A., Lodochnikova O.A., Mamedova V.L., Korshin D.E., Mamedov V.A. (2019). Intermolecular head-to-head interaction of carbonyl groups in bicyclic hydrogen-bonded synthon based on β-hydroxy ketones. CrystEngComm.

[30] Wu Y., Liao L.D., Pan H.C., He L., Lin C.T., Tan M.C. (2017). Fabrication and interfacial characteristics of surface modified Ag nanoparticle based conductive composites. RSC Adv..

[31] Garcia-Gavilan C.M., Lopez-Vega C., Mendez-Sanchez I.M. (2017). Ulcerative colitis with gastric and duodenal involvement. Rev. Esp. Enferm. Dig..

[32] Sasaki M., Okada K., Koyama S., Yoshioka U., Inoue H., Fujiyama Y., Bamba T. (1996). Ulcerative colitis complicated by gastroduodenal lesions. J. Gastroenterol..

[33] Horje C.S.H.T., Meijer J., Rovers L., Van Lochem E.G., Groenen M.J.M., Wahab P.J. (2016). Prevalence of Upper Gastrointestinal Lesions at Primary Diagnosis in Adults with Inflammatory Bowel Disease. Inflamm. Bowel Dis..

[34] Lin J., McKenna B.J., Appelman H.D. (2010). Morphologic findings in upper gastrointestinal biopsies of patients with ulcerative colitis: A controlled study. Am. J. Surg. Pathol..

[35] Sun Y., Zhang Z., Zheng C.-Q., Sang L.-X. (2021). Mucosal lesions of the upper gastrointestinal tract in patients with ulcerative colitis: A review. World J. Gastroenterol..

[36] San Aye K., Lin Htun L., Win T.M., Aye M.T., Tin D., Aye T.T. (2021). Gastroduodenal Ulcerative Colitis in Association with Ulcerative Pancolitis. Case Rep. Gastrointest. Med..

[37] Yang Y., Li C.Q., Chen W.J., Ma Z.H., Liu G. (2020). Gastroduodenitis associated with ulcerative colitis: A case report. World J. Clin. Cases.

[38] Li M., Liu Y., Cui J., Qin H., Shi Y., Zhang S., Zhao Y. (2019). Ulcerative colitis with mucosal lesions in Duodenum: Two case reports. Medicine.

[39] Nomura Y., Moriichi K., Fujiya M., Okumura T. (2017). The endoscopic findings of the upper gastrointestinal tract in patients with Crohn’s disease. Clin. J. Gastroenterol..

