# Supplementary material for: The Influence of the Hydrophobic Polymeric Coating on 5-ASA Release from the Bipolymeric Milibeads with Amidated Pectin
Source: Materials (Basel). 2021 Jul 14;14(14):3924. doi: 10.3390/ma14143924 (PMC8306472; doi:10.3390/ma14143924)
Supplement: Supplementary file 1 [file materials-14-03924-s001.zip › materials-1261951-SI.pdf]

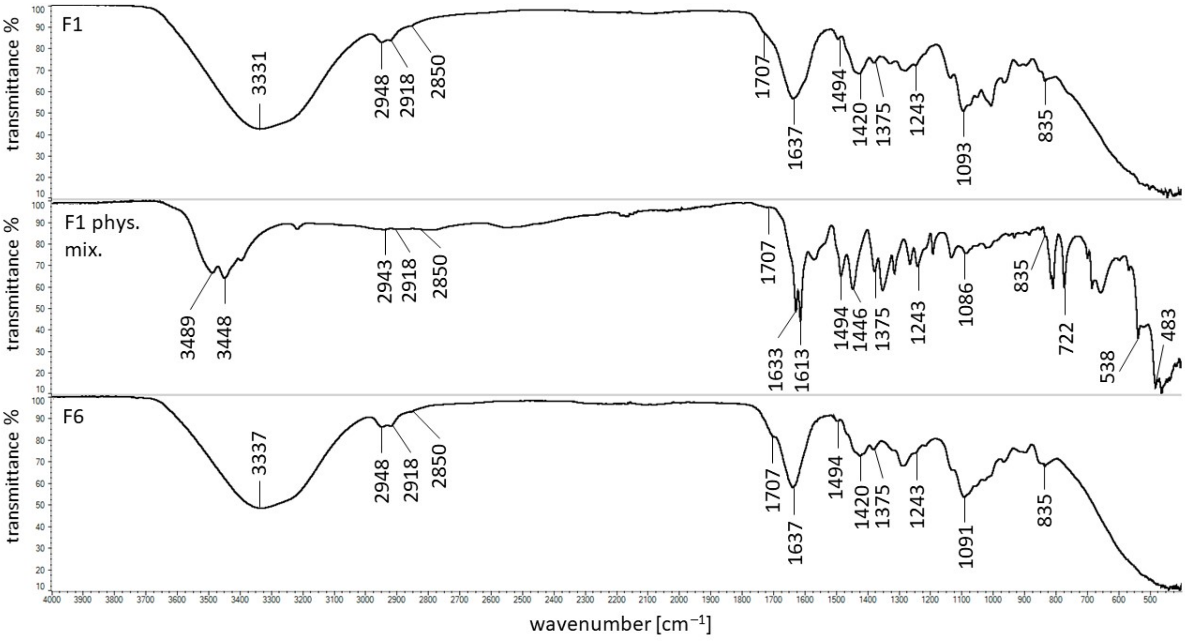

Figure S1. FTIR spectra of formulation F1, its physical mixture, and F6.

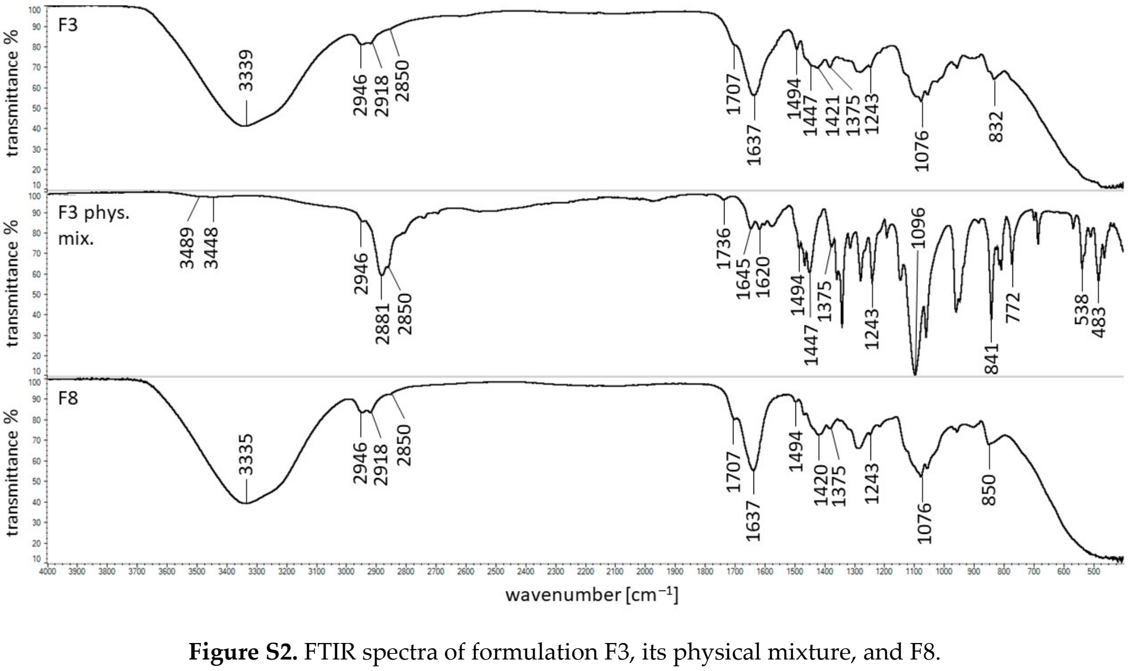

Figure S2. FTIR spectra of formulation F3, its physical mixture, and F8.

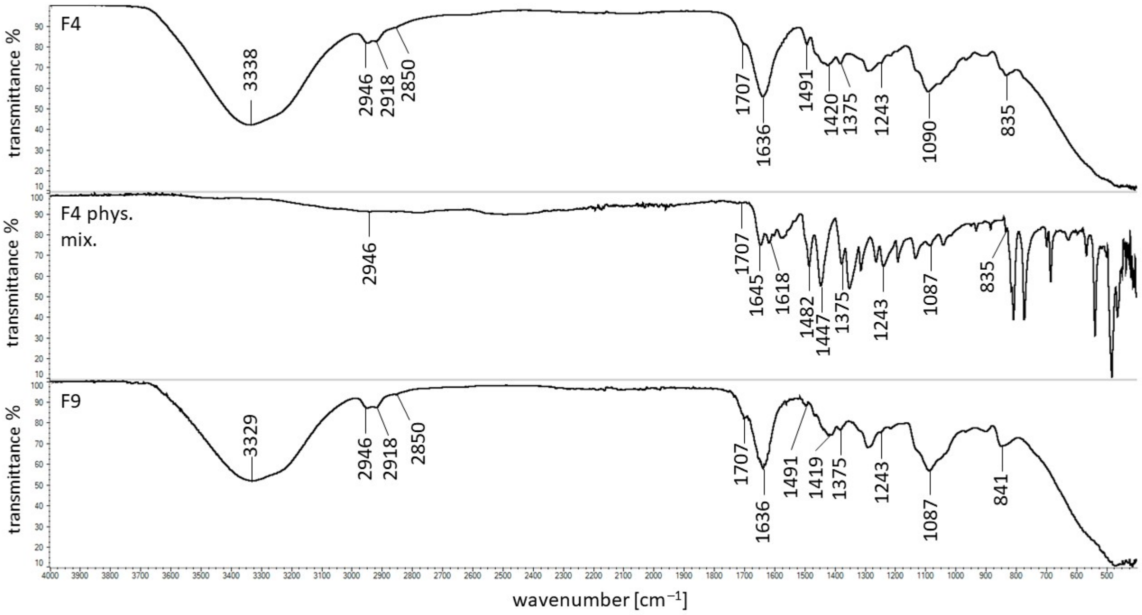

Figure S3. FTIR spectra of formulation F4, its physical mixture, and F9.

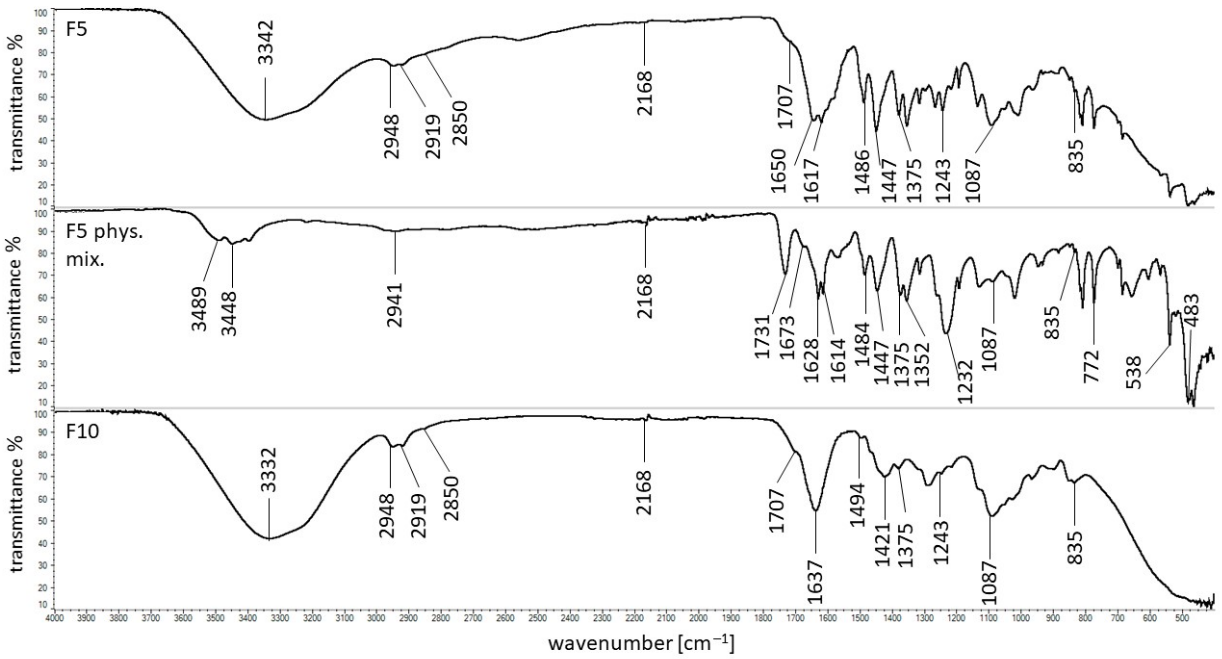

Figure S4. FTIR spectra of formulation F5, its physical mixture, and F10.

DSC Investigation—Method

A differential scanning calorimeter (DSC 214 Polyma, Netzsch, Selb, Germany) was employed to measure the DSC thermograms of F1–F10 beads and the corresponding physical mixtures of their ingredients. The samples of 3–5 mg were scanned in closed aluminum crucibles. The study was carried out in the temperature range 10 to 300 °C. The heating velocity was 5 K/min, and the flow rate of nitrogen was 50 mL/min.

DSC Investigation-Results and Discussion

The DSC study was conducted to characterize the thermal differences occurring due to interactions between the components of the beads. The thermal investigation of uncoated beads was described in the previous work [12]. In the present research, we focused on the possibility of forming a bond between the coating and the components of the formulations. The recorded maxima at the corresponding temperature values were listed in Table S1. The thermograms of the coating together with curves of F1, F6, and the physical mixture of F1 (M_{F1}) were shown in Figure S5. The thermograms of the rest formulations with the physical mixtures were shown in Figures S6–S9. On the thermogram of PVAC-D, one endotherm situated at 178.1 °C and one exotherm at 245.9 °C were found.

The endotherm of APN at 73 °C connected with the evaporation of water was present in the range of 71.0–91.3 °C in the obtained thermograms. However, the maximum was not observed in the curves of physical mixtures M_{F1}–M_{F5} because they were overlapped by strong and broad signals from $CaCl_2$.

Table S1. The DSC endotherms and exotherms (↓) of formulations F1–F10 and the corresponding physical mixtures (M). The listed numbers refer to the value of temperature.

	**F1**	**F6**	**M_{F1}**	**F2**	**F7**	**M_{F2}**	**F3**	**F8**	**M_{F3}**	**F4**	**F9**	**M_{F4}**	**F5**	**F10**	**M_{F5}**
APN	86.1	88.6	—	86.7	—	—	93.6	71.0	—	71.2	73.2	—	—	—	91.3
APN	167.1	162.2	168.3	164.5	159.0	157.8	167.0	167.9	178.9	163.9–167.6	165.7	174.7	166.5	160.2	165.4
APN	186.9	190.1	—	195.6	188.3	190.0	189.7	182.5	195.5	197.5	193.6	192.8	199.1	195.6	196.7
$CaCl_2$	—	—	102.4	—	—	107.0	—	—	96.7	—	—	97.0	—	61.9	91.3
$CaCl_2$	—	—	129.8	—	—	132.0	—	—	128.5	—	—	126.8	—	—	98.7
PVAC-D	135.8	139.7	147.1	139.1	131.7	157.8	138.1	139.7	171.9	143.9	137.9	158.7	137.3	141.1	121.3
PVAC-D/APN	239.6 (↓)	249.3 (↓)	241.9 (↓)	239.2 (↓)	232.5 (↓)	—	229.2 (↓)	227.2 (↓)	213.9 (↓)	246.1 (↓)	241.6 (↓)	223.3 (↓)	232.3 (↓)	—	224.3 (↓)
5-ASA	260.1	—	235.6	259.6	—	237.7	265.1	—	234.1	256.3	—	243.7	259.7	—	250.1
PA	—	—	—	225.9	218.3	222.7	—	—	—	—	—	—	—	—	—
PEG	—	—	—	—	—	—	—	—	60.1	—	—	—	—	—	—
AX	—	—	—	—	—	—	—	—	—	126.8 (↓)	117.4 (↓)	108.1 (↓)	—	—	—
AX	—	—	—	—	—	—	—	—	—	136.0	—	—	—	—	—
AX	—	—	—	—	—	—	—	—	—	—	144.5	—	—	—	—
AX	—	—	—	—	—	—	—	—	—	203.0 (↓)	200.6 (↓)	—	—	—	—
AX	—	—	—	—	—	—	—	—	—	244.9	—	—	—	—	—
PVAC-P	—	—	—	—	—	—	—	—	—	—	—	—	—	—	39.3

The maxima of APN at 178.9 and 192.8 °C were noticed in the thermograms of F1–F10 and the physical mixtures M_{F1}–M_{F5}, although they were shifted toward lower temperatures direction. The exothermic process of APN at 236.2 °C was found in the temperature range of 213.9–249.3 °C almost on all curves. However, the signal belonging to PVAC-D was present very close, at 245.9 °C, and it was difficult to assign the maxima unambiguously to APN or PVAC-D. The broad endotherm of PVAC-D at 178.1 °C was also near the peak of APN, although based on the shape of the signals, the maxima were assigned to the coating polymer. The endotherms belonging to PVAC-D were shifted to lower temperatures direction and were found between 121.3–171.9 °C. The signal of 5-ASA was noticed in thermograms of F1–F5 as well as their physical mixtures M_{F1}–M_{F5}, meaning the lack of interaction between the drug and the rest of the ingredients. The maxima of $CaCl_2$ were found

mainly in the thermograms of physical mixtures M_{F1}–M_{F5}. They were not observed in the thermograms of formulations F1–F10 due to the interaction of Ca^{2+} with APN. It was interesting that in the thermal plots of F2, F7, and M_{F2} doped with PA, the signal of PA at 62.4 °C was not monitored, although the peak at 221.5 °C was observed between 218.5 and 225.9 °C. The absence of the maximum at 62.4 °C may be caused by the evaporation of water molecules. The sharp maximum of PEG 4000 was observed in the curve of M_{F3} at 60.1° C but was not found in the thermal profile of F3 nor F8, suggesting the interaction with polymers. The AX exothermic event at approximately 122.1 °C was observed in the range of 108.1–126.8 °C in F4, F9, and M_{F4} thermograms. The maxima at 182.3 °C were present in the curves of F4 and F9 in the range of 200.6–203.0 °C. The signals were complex, and it was difficult to assign the peaks to the components unambiguously. The sharp endotherm of PVAC-P at 47.1 °C was present only in the thermogram of M_{F5}, although it disappeared in the curves of formulations F5 and F10 doped with PVAC-P.

The DSC investigation revealed that the lack of some peaks of synthetic polymers in the thermograms might indicate the interaction with another ingredient of the formulation. It is consistent with the results obtained from the release study. The calculated kinetic parameters were different for the release of 5-ASA from all formulations. Additionally, the obtained values of parameters f_1 and f_2 as well as statistical analysis indicated the differences in the release of the drug from formulation F2 in comparison to the release from F1, F3–F5. FTIR results also suggested the interaction between PA and the coating of the beads.

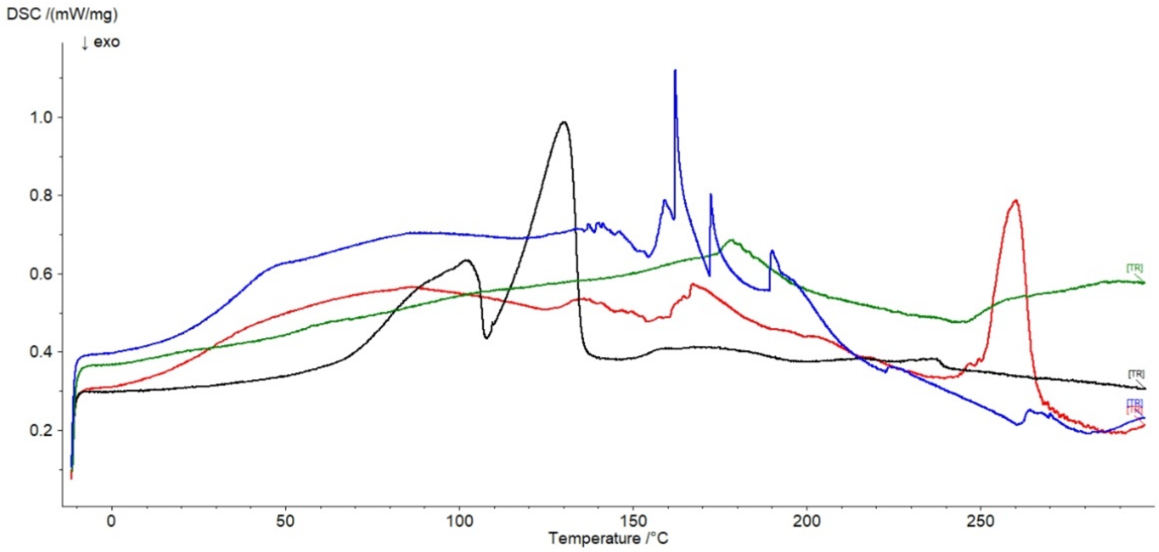

Figure S5. The thermogram of formulation F1 (red line), F6 (blue line), and the physical mixture of F1 (black line) as well as the curve of PVAC-D (green line).

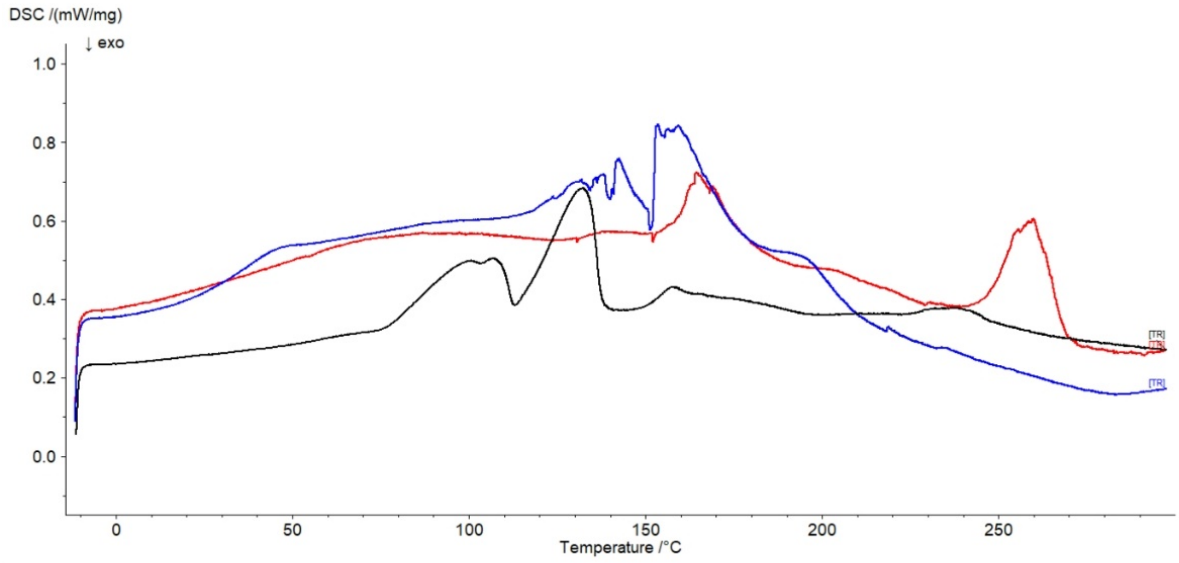

Figure S6. The thermogram of formulation F2 (red line), F7 (blue line), and the physical mixture of F2 (black line).

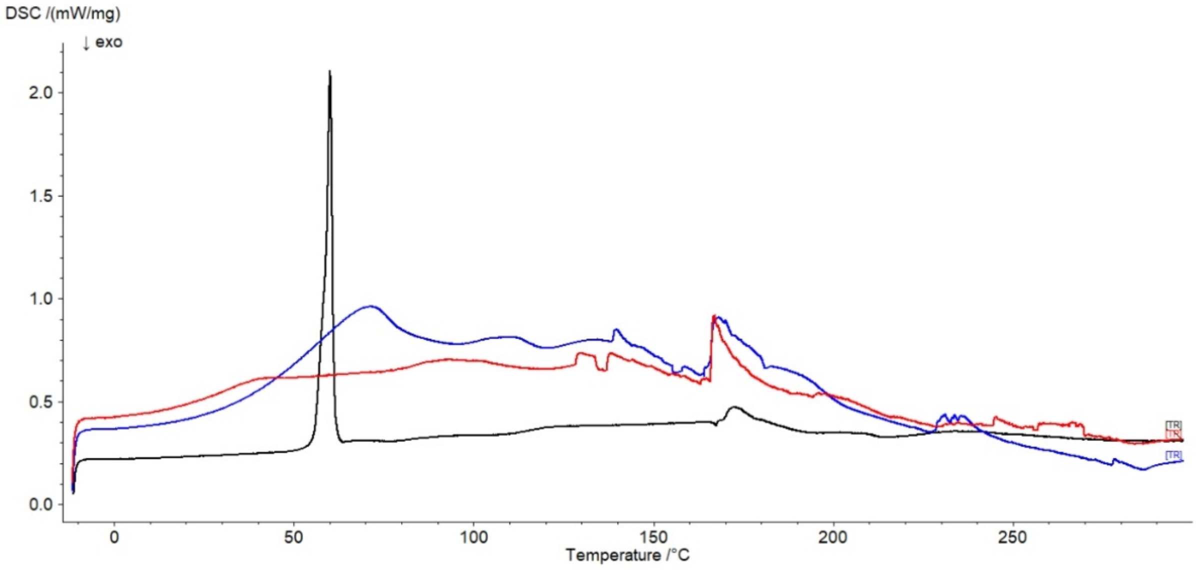

Figure S7. The thermogram of formulation F3 (red line), F8 (blue line), and the physical mixture of F3 (black line).

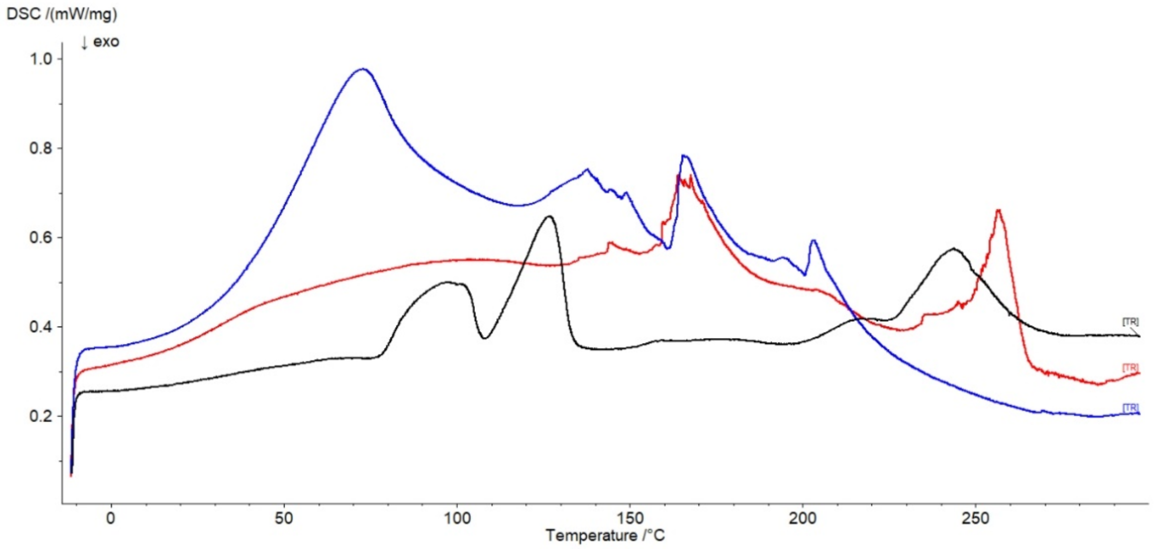

Figure S8. The thermogram of formulation F4 (red line), F9 (blue line), and the physical mixture of F4 (black line).

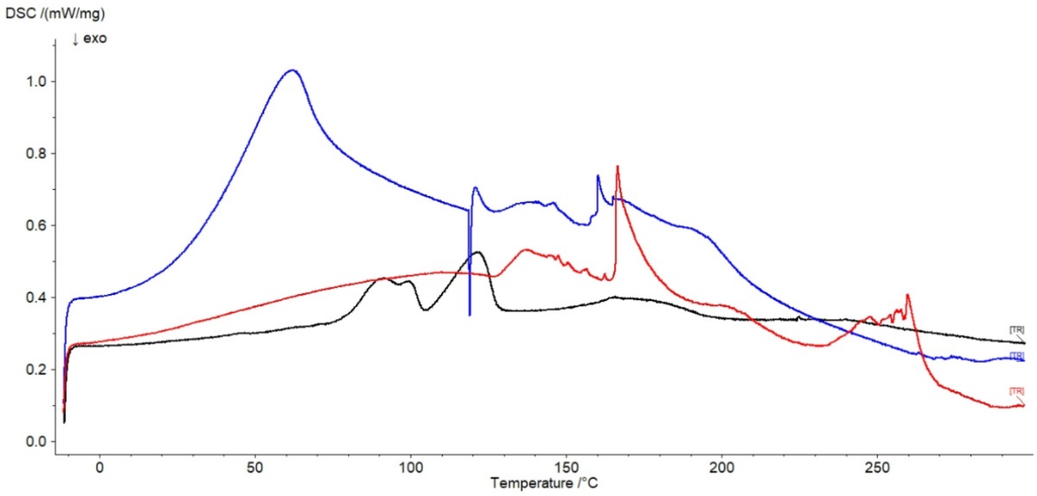

Figure S9. The thermogram of formulation F5 (red line), F10 (blue line), and the physical mixture of Figure 5 (black line).
